# The social construction of Aduhelm in the context of pharmaceutical ambiguity: exploring narratives from informal caregivers, medical professionals, and redditors on r/Alzheimers

**DOI:** 10.3389/fsoc.2025.1636160

**Published:** 2026-01-05

**Authors:** Zack Simoni, Victoria Hilfiker

**Affiliations:** Department of Sociology, Anthropology, and Geography, University of Tennessee at Chattanooga, Chattanooga, TN, United States

**Keywords:** medical sociology, Alzheimer’s disease, informal care givers, attitudes toward pharmaceuticals, pharmaceutical ambiguity, qualitative research, pharmaceuticalization, Aduhelm

## Abstract

**Introduction:**

In 2021, the Food and Drug Administration approved Aduhelm (Aducanumab), a pharmaceutical developed to treat Alzheimer’s disease. At first, many in the advocacy and caregiving community responded with jubilation as there are currently few effective treatments to reduce or eliminate the symptoms of Alzheimer’s disease. However, suspicions about the approval process quickly arose as well as concern from the medical community, which led to controversy about the drug. With this context in mind, our study aims to analyze the viewpoints of caregivers, medical professionals, and Redditors on a popular subreddit for caregivers, as the controversy unfolded. Understanding caregivers’ perspectives is vital, as they play an important role in medical compliance and Aduhelm’s reception may impact adherence to future treatments. We aim to address two research questions: (a) What are the attitudes towards Aduhelm of caregivers, medical professionals, and members of an online forum associated with caregiving for Alzheimer’s patients (r/Alzheimers) and (b) How does the controversy surrounding Aduhelm influence attitudes about the health system and medical practice?

**Methods:**

We conducted a grounded theory analysis of 23 semi-structured interviews with caregivers and five medical professionals, alongside an online discourse analysis of r/Alzheimers.

**Results:**

Our findings reveal various concerns about Aduhelm involving efficacy, safety, and affordability. Most notably, we find these narratives increased a sense of medical mistrust from participants, which may be problematic for adherence and the doctor/patient interaction.

**Discussion:**

Drawing on sociological literature, we introduce the concept of pharmaceutical ambiguity, a theoretical framework for understanding these social phenomena. This study highlights how controversies like that surrounding Aduhelm can deeply erode trust in medical systems.

## Introduction

Alzheimer’s disease is a progressive, dementia-related condition that gradually impairs cognitive abilities, including memory, thought processes, and language control. It is the most prevalent form of dementia in the United States, affecting approximately 6.7 million Americans in 2023 (CDC, 2023; Rajan et al., 2021). The disease imposes various challenges on caregivers, such as stigma, emotional distress, financial burden, and caregiver burnout (Del-Pino-Casado et al., 2021; Sheehan et al., 2021). Considering the significant societal and personal effects of Alzheimer’s disease, there have been numerous attempts at medical interventions aimed at reducing the progression of the disease. However, existing treatments show limited efficacy at reducing the progression of the debilitating disease.

Bearing these challenges in mind, pharmaceutical companies are engaged in a billion-dollar race to patent, manufacture, and market medications aimed at slowing or reversing the progression of Alzheimer’s disease (Tampi et al., 2021). One such company, Biogen, claimed their drug, Aduhelm, slowed the disease’s progression. Initially, some in the media, caregiving community, and advocacy groups proclaimed Aduhelm was a breakthrough, but this optimism was short-lived as the drug faced severe criticism due to concerns about the approval process (Tampi et al., 2021). As a result of the unprecedented controversy, Biogen announced the discontinuation of Aduhelm in January of 2024, with access for existing patients ceasing in November 2024.

Considering the Aduhelm controversy, researchers must understand how patients, caregivers, and medical professionals conceptualize the legitimacy of pharmaceutical products, as this understanding may affect compliance with future pharmaceutical innovations, the doctor-patient interaction, attitudes about the health system, and the general well-being of all individuals involved. However, few qualitative studies have examined how caregivers of Alzheimer’s disease patients and medical professionals navigate the moment of uncertainty brought about by the Aduhelm controversy.

To address this gap, our paper aims to answer the following research questions: (a) What are the attitudes toward Aduhelm by caregivers, medical professionals, and members of an online forum associated with caregiving for Alzheimer’s patients (r/Alzheimers) and (b) How does the controversy surrounding Aduhelm influence attitudes about the health system and medical practice? We begin with a review of pertinent literature.

## Review of literature

### Caregiving for individuals with Alzheimer’s disease and the controversial approval of Aduhelm

Informal caregivers refer to those who provide care, generally in the home environment, for an aging parent, spouse, other relative, or unrelated person, or for an ill, or disabled person. Due to the progressive nature of Alzheimer’s disease and its impact on cognitive functioning, caregivers often make medical decisions regarding care and treatment. Although not all caregiving experiences are the same, an abundance of research identifies an increased likelihood of “caregiver burnout,” due to stressors associated with caregiving for individuals with Alzheimer’s disease (del-Pino-Casado et al., 2021). Among various stressors, caregivers may express feelings of guilt, resentment, and sadness, in addition to markedly elevated levels of psychological distress, particularly depression and anxiety (Sheehan et al., 2021). Caregivers also may encounter numerous financial challenges trying to afford treatments, the cost of long-term care living, and lost wages due to missing work to care for an individual with Alzheimer’s disease (Willert and Minnotte, 2021). Furthermore, informal caregiving may stress social relationships within the family as caregiving may incur a heavy cost for some family members and not others (Kılıç et al., 2024).

Despite challenges faced by caregivers, there are currently limited effective treatment options for Alzheimer’s disease. Thus, the desperation for any kind of treatment to ameliorate symptoms associated with Alzheimer’s disease is at the forefront of many caregivers’ and medical professionals’ minds (Kılıç et al., 2024). Pharmaceutical companies are well aware of this untaped market and have responded by prioritizing development of drugs aimed at reducing symptoms associated with Alzheimer’s disease, if not reversing the course of Alzheimer’s disease entirely (Woloshin and Kesselheim, 2022). One drug with such lofty goals in mind, Aducanumab (Aduhelm), was licensed by the German company Biogen in 2007. By the data collection phase of this paper, Biogen had received FDA approval to sell the drug. However, there were many complicated challenges ahead for the drug. We address that history below.

Initial trials of Aduhelm (PRIME, phase 1b) began in 2012 with 166 participants reporting mild Alzheimer’s disease related symptoms. In 2015, an analysis from Biogen showed Aduhelm reduced amyloid deposition in six cortical regions of the brain, a theoretical factor in Alzheimer’s-related cognitive decline (Woloshin and Kesselheim, 2022). In October 2019, another analysis indicated significant clinical benefits at high doses of Aduhelm. Biogen quickly submitted a biologics license application to the FDA, and clinical trials resumed in 2020. Despite an FDA advisory committee voting against its approval in November 2020, the FDA approved Aduhelm under the accelerated approval pathway in June 2021.

The approval initially received enthusiastic support from the media, caregivers, advocacy groups, Wall Street, and Biogen itself as numerous articles expressed optimism about the findings (Liu, 2021). However, the medical community quickly raised concerns about the approval process’s integrity, leading to intense scrutiny of the process. As a result, several FDA advisory committee members resigned in protest highlighting unethical practices within the accelerated approval process (Mahase, 2021).

Events reached a turning point in January 2022 when the Centers for Medicare and Medicaid Services (CMS) decided to cover Aduhelm only for patients in clinical trials, citing the need for more evidence on its efficacy and safety (Woloshin and Kesselheim, 2022). For a short time, Aduhelm remained available only through clinical trials via CMS, and Biogen has since shifted focus to other similar drugs (see Lecanumab). As of January 2024, Biogen announced the discontinuation of Aduhelm, with access for existing patients ceasing in November 2024. This latest development underscores the drug’s controversial history and the period of pharmaceutical ambiguity examined in this study. Our data were collected during this critical period, from Fall 2021 to Spring 2022, when the drug was actively on the market and faced both jubilation as well as profound uncertainty.

### The construction of pharmaceuticals, Pharmaceuticalization, and medical uncertainty

Sociological research on pharmaceuticals has drawn attention to how pharmaceuticals are understood and used in everyday life. Pharmaceuticals are constructed not only with technical meanings involving their biomedical functions, but also with strong social and cultural narratives for how they should be used and under what circumstances (Conrad, 1985; Gabe et al., 2016; Hodgetts et al., 2011). As Cohen et al. (2001) note, their use is shaped by cultural repertoires, social relationships, the medical condition being experienced, and the identities of their consumers (Dew et al., 2014; Lumme-Sandt et al., 2000). Furthermore, in part due to a shift toward consumer-based medicine, users are increasingly recognized as being knowledgeable actors, assessing the risks and benefits and making informed choices about medicine use drawing on what Webster et al. (2009) call “lay pharmacology.” Choices are often made in consultation with professionals but in most cases various forms of information channels pay a role, particularly lay social networks and social media (Will and Weiner, 2015).

One article particularly relevant to this study, from Shoemaker and Ramalho de Oliveira (2008), used a phenomenological approach to describe the meaning making process of pharmaceuticals (the medication experience) and found 4 key themes that influenced adherence to a pharmaceutical regimen. The four themes of the medication experience include the initial encounter, which leads to questioning the medication; the bodily effects of medications or weighing the pros and cons; unremitting nature or ongoing need to take medications; and the ways in which patients exert control over their medication regimens. Thus, it is vital to note that the social construction of pharmaceuticals can affect the patient’s experience as well as compliance and adherence to pharmaceutical medication.

It is also important to note that the construction of pharmaceuticals takes place within a larger social context nestled within a neoliberal, consumer-driven model of healthcare, amid the ever increasing effects of pharmaceuticalization (Figert, 2011; Gabe et al., n.d.). Pharmaceuticalization identifies the process whereby social, behavioral, or bodily conditions are primarily treated with pharmaceutical intervention (Abraham, 2010). It means that pharmaceuticals are often framed as the best solution to medical problems and an ever-increasing number of social problems are framed in this light as opposed to models of community care, holistic or integrative medicine approaches. As Joan Busfield puts it, there is a “pill for every ill” (Busfield, 2006, 2010).

The pharmaceutical industry plays a significant role in the pharmaceuticalization process by medicalizing conditions, while also investing heavily into marketing research, direct to consumer advertising, and lobbying political leaders (Barker, 2011; Williams et al., 2011). Once approved, drugs are marketed to healthcare professionals and the public, shaping prescribing practices and public perceptions (Busfield, 2006; DeJong et al., 2016). Thus, the rise of pharmaceuticalization within the context of a consumer-driven health system requires consumer trust in various stakeholders, including the pharmaceutical industry, government, regulatory agencies, and doctors (Figert, 2011). Although pharmaceuticals have become the dominant form of treatment in the United States for a litany of diseases and disorders, there remains a large degree of medical uncertainty in terms of caregiving for those with Alzheimer’s disease and deciphering the efficacy of various pharmaceutical treatment options.

In terms of the sociological literature regarding medical uncertainty, research from Han et al. (2011), found that caregivers of Alzheimer’s disease patients must learn to manage uncertainty stemming from limitations in medical research and knowledge alongside the ever-changing understanding of Alzheimer’s pathology. Furthermore, the effectiveness of new treatments contribute to this uncertainty and it means families and patients face convoluted information regarding the diagnosis of Alzheimer’s (Swallow, 2020). Most notably, individuals diagnosed with mild cognitive impairment show signs of cognitive decline that exceed “normal aging,” yet may not develop full-blown Alzheimer’s disease, leading to uncertainty about the future (Swallow, 2020). Furthermore, there is also uncertainty due to the unpredictable nature of the disease and when pharmaceutical intervention becomes warranted (Mackintosh and Armstrong, 2020).

Lastly, research from Czarnecki et al. (2023) characterize ways in which sociopolitical contexts can create an external source of uncertainty. According to their work on the Dobbs decision, Czarnecki et al. argue the sociopolitical context may include legal, regulatory, and policy factors that shape the organization and delivery of healthcare. Uncertainty stemming from the complex interpretation and implementation of the Dobbs decision caused stricter implementation of regulations than the laws necessitate and provided fertile ground for misinformation. For example, they note that uncertainty about the legality of abortion increased during periods of legislative activity, with some people believing abortion was illegal even when it wasn’t. They also found that terms related to abortion (like “lethal” and “life-threatening”) can be ambiguous and not in line with clinical realities, which can foment misinformation and confusion. As such, macro-level forces including pharmaceuticalization and the role of various stakeholders may impact the social construction of pharmaceuticals and affect medical decisions. Together, these insights from sociological literature and the controversy surrounding Aduhelm show how uncertainty may be produced and is shaped by structural forces external to medicine, as opposed to originating from within.

### Previous research regarding attitudes about Aduhelm

Qualitative studies about the attitudes of informal caregivers regarding Aduhelm are few and far between. However, one quantitative study from Zissimopoulos et al. (2022) analyzed the general public’s attitudes toward the then newly FDA-approved Alzheimer’s drug just after its controversial approval in June 2021. Despite the high-profile approval and subsequent publicity surrounding Aduhelm, the survey revealed a notable lack of understanding of the drug among lay Americans. The public did not show a broad understanding of potential impacts or a significant interest in pursuing it as a treatment option. One thing to keep in mind is that the Zissimopoulos study was largely interested in the general public’s attitudes about Aduhelm and not caregivers, those who would, presumably, have more knowledge about Aduhelm.

Another quantitative study from Dhruva et al. (2023) surveyed medical professionals’ attitudes toward the FDA’s accelerated approval of Aduhelm. They found that neurologists demonstrated significant skepticism, and the hesitation was predominantly rooted in concerns over the strength of evidence supporting its approval. Notably, most neurologists did not view approval as justified by the existing data, indicating a prevalent view that commercial interests may have more driven the approval rather than robust scientific validation. 86% of their participants said they could not prescribe or recommend Aduhelm, and 67% of neurologists reported losing trust in other drugs approved through the accelerated approval program due to the FDA’s decision with Aduhelm. Although these studies are vital for the sociological literature, they do not provide an understanding of the motivations and meaning making process of Aduhelm, a task which qualitative research is designed to identify and this paper hopes to address.

In summation, the relevant sociological literature above highlights several key factors situating this paper. First, informal caregivers face a significant burden, grappling with emotional, financial, and decision-making challenges, leading them to sincerely desire relief for their loved ones. Second, the controversial approval of Aduhelm, marked by conflicting scientific evidence and concerns about the FDA’s process, exemplifies the broader phenomenon of medical uncertainty. These studies are further contextualized within the framework of pharmaceuticalization and the social construction of pharmaceuticals, where drugs are increasingly constructed as primary solutions to medicalized conditions and social problems. Third, although there is an abundance of research geared toward studying the construction of pharmaceutical and compliance to drug regimens, there is a gap in the literature regarding how caregivers and medical professionals make sense of medical uncertainty as it pertains to novel pharmaceutical medications aimed at addressing Alzheimer’s disease, and the approval of Aduhelm in particular.

## Materials and methods

Grounded Theory is an inductive systematic research method used to identify and provide insight into patterns of meaning-making in the description of social phenomena (Glaser and Strauss, 2017). A grounded approach emphasizes the “constant comparative method” where data collection and analysis occur simultaneously, allowing emerging concepts to shape further data gathering. In other words, the goal is to generate a theory that is “grounded” in the data, meaning it accurately reflects the phenomena being studied (Glaser and Strauss, 2017). We chose to use grounded theory because it provides flexibility for interpreting emerging themes and exploring new social contexts. Beyond simply identifying patterns in behaviors, this approach also enables the researcher to uncover ways of knowing and experiencing behaviors as well as their motivations (Sundler et al., 2019). Likewise, the data driven approach allows researchers to utilize theoretical sampling to fully address any emerging theoretical insights. Thus, this approach fulfilled the goal of fully understanding experiences, meaning, and attitudes regarding Aduhelm within the context of the controversy.

### Data collection

The recruitment process primarily utilized Reddit, a digital media platform known for its diverse content and community discussions. Redditors (community members) rate content through upvotes and downvotes, enhancing visibility for posts and comments. The study focused on r/Alzheimers, a subreddit or online community, dedicated to caregiving for Alzheimer’s disease, which had over 14,000 users in 2023. Initially, the researchers chose Reddit due to the Covid-19 pandemic constraints and Reddit’s access to diverse populations for qualitative and quantitative research (Draucker et al., 2007; Jamnik and Lane, 2017).

The lead author posted a recruitment script on r/Alzheimers, outlining the inclusion criteria and other pertinent information. Participants needed to be current or former caregivers of individuals with Alzheimer’s disease, aged 18 to 75. The script was posted four times, resulting in 23 caregiver participants when data saturation was reached. Via theoretical sampling (Draucker et al., 2007), the sample expanded to include early-stage Alzheimer’s caregivers and medical professionals, including a primary care physician, a medical resident, and three neurologists. Medical professionals were recruited in much the same way as caregivers in that a script was posted to similar subreddits geared toward these groups, namely r/neurology and r/doctors. Although the focus of the study is caregivers, the inclusion of medical professionals helped to substantiate claims from caregivers and further build a coherent theory.

Semi-structured interviews were the primary data collection tool among caregivers and medical professionals, guided by questions and probes designed to explore the caregiving experience and attitudes toward treatment. During phone conversations, participants discussed symptom recognition, health system engagement, respite care, online social support, social role adjustments, and attitudes toward medication. Specific questions and probes emerged to ask about their perspectives and experiences with Aduhelm. Data saturation was reached after 23 interviews with caregivers and 5 interviews with medical professionals ensuring no new themes emerged, justifying the sample size (*N* = 28). According to Strauss and Corbin (1997), saturation is reached when new data no longer generates new properties, dimensions, or relationships. Instead, new data simply confirms what was established from old data. There is not a clear cut-off in terms of how many interviews are required. However, researchers Guest et al. (2006) used an experimental research design to demonstrate that the threshold for theoretical saturation can occur after as few as 12 interviews. Considering our study entailed 28 interviews, we believe the sample size allowed us to find patterns and thoroughly answer the research question. In practice, we determined saturation was achieved when roughly three consecutive interviews yielded no fundamentally new codes, categories, or novel relationships between existing concepts, thereby justifying the final sample size. To gather basic demographic information about our sample, a short online survey accompanied the interviews (See Tables 1, 2).

**Table 1:** Demographics of informal caregiver (*N* = 23).

Variable	Frequency
Race	
White	19
Black	2
Asian	1
Other	1
Sex	
Male	10
Female	12
Non-Binary	1
Age	
25–35	2
36–45	6
46–55	6
Over 55	9

**Table 2:** Demographics of medical professionals (*N* = 5).

Variable	Frequency
Race	
White	3
Black	0
Asian	2
Other	0
Sex	
Male	4
Female	1
Non-binary	0
Age	
25–35	0
36–45	1
46–55	4
Over 55	0

We also used triangulation and expanded the sample to include redditors in the subreddit, r/Alzheimers, which is primarily composed of caregivers. Triangulation in qualitative research includes the use of multiple data sources to validate findings, enhancing the credibility and robustness of the results (Flick, 2004). In this case, we conducted an online discourse analysis of the r/Alzheimer’s subreddit using a methodological approach guided by work from Recuber (2017). Via the memoing process, a pattern emerged in that the caregivers were using Reddit as a spot to find relevant information and seek support. We then decided to begin the online discourse analysis around the 5^th^ interview with caregivers. We used the search tool on the subreddit to limit posts and replies that included the terms “Aduhelm,” “Aducanumab,” and “Biogen.” Posts and replies using the terms began in the summer of 2021, shortly after the FDA granted accelerated approved. Some posts were submitted to reddit before the beginning of this study, but the majority were posted as the interviews were being conducted. So, most of the posts were treated as new. As such, the researchers consistently checked the subreddit for posts and replies containing the search terms. Although Reddit has an upvote feature, the researchers used all the posts and comments as data and did not distinguish based upon upvotes. In total, this included 40 unique posts and 314 replies or comments to those posts.

### Analysis

Analysis of the qualitative data (both interviews and online discourse) consisted of “line by line” initial coding. Initial codes were gradually refined to form more precise categories or concepts, and through further refinement, initial themes (Strauss and Corbin, 1990; Strauss and Corbin, 1997). As well as the lead author, an undergraduate honors’ student aided in the coding process to improve inter-coder reliability. Each investigator independently coded the first five interviews and compared codes to create a preliminary code list. The code list remained flexible as we evaluated the validity of emerging themes and concepts while discussing themes to assure reliability within the data.

**Figure 1:**
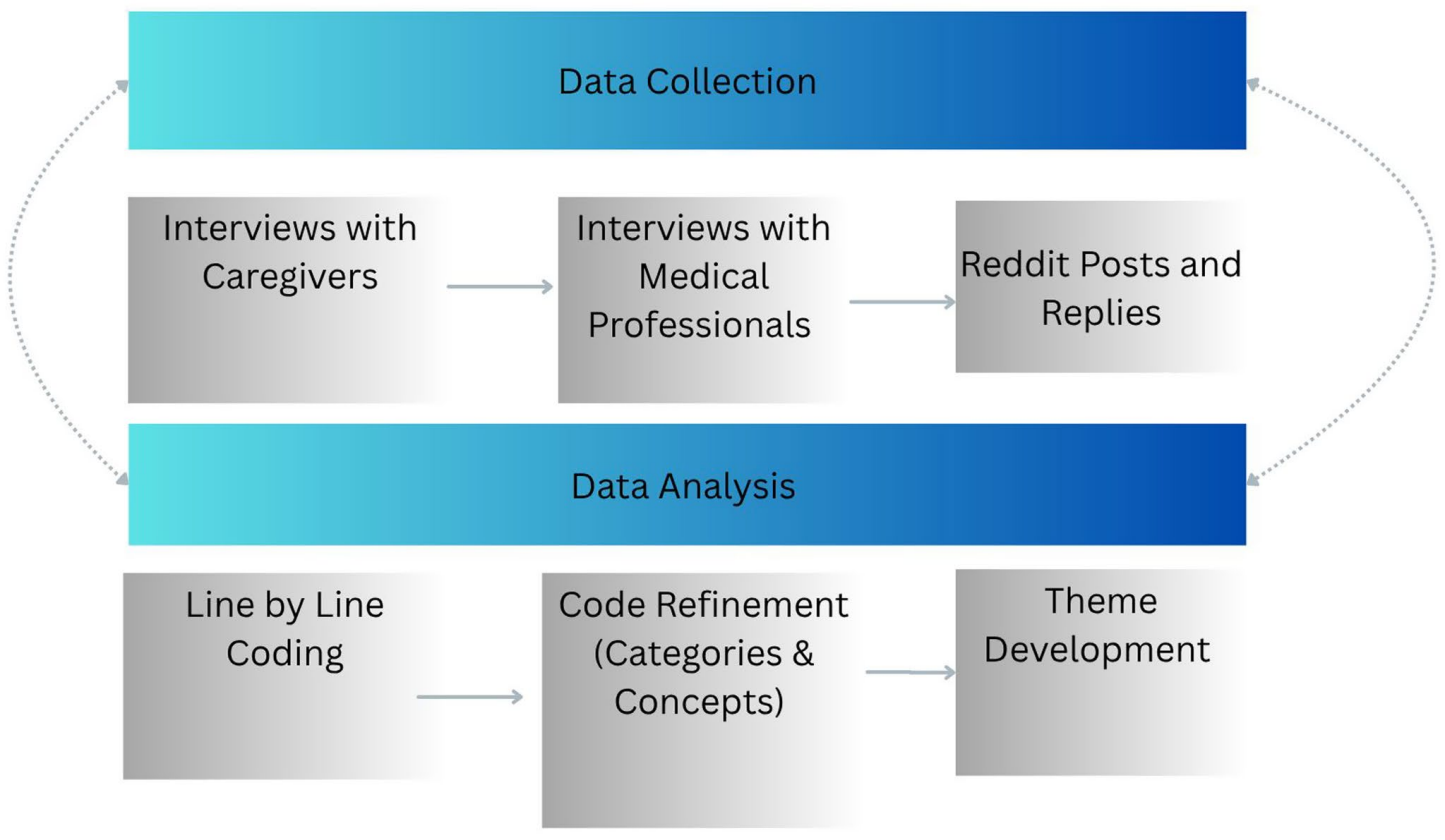
Qualitative data analysis process.

Using constant comparison, codes and emerging themes were analyzed to see if they worked or “fit” in relation to the coded extracts, and the entirety of the data. Links between themes were explored revealing both the meaning of the themes as well as their relationship with other codes, categories, and other themes (Strauss and Corbin, 1997). Memo writing was also used throughout both the data collection and analysis process to help clarify concepts, make connections between various concepts, account for reflexivity (Palaganas et al., 2017), while also allowing the researchers to elevate codes into themes (Strauss and Corbin, 1997). As is standard practice within grounded theory research, data collection and data analysis occurred simultaneously. We used this analytical strategy to analyze both the semi-structured interview data as well as the online discourse analysis of the subreddit. Via triangulation, data from the discourse analysis was compared to data from the interviews. Qualitative data software NVivo 12 aided in the management of transcripts and reddit posts to organize categories into themes and ensure consistency in coding. This study received institutional review board approval abiding by the ethics procedures of our institution. For a visual representation of the data collection and data analysis processes, see Figure 1.

With the qualitative nature of this study in mind, it is essential to explore the author’s standpoint via the practice of reflexivity. In short, the goal of reflexivity is to describe an individual’s overall perspective and the relationship they hold toward a research project (Holmes, 2020; Savin-Baden and Major, 2013). This includes the researcher’s presuppositions, any potential personal impact, and preconceived notions. These issues could potentially guide the researcher’s interactions with participants and the research context, specifying how, when, where, and to what extent these facets could influence the study’s development. Thus, researchers must critically examine their own viewpoints and/or knowledge and explain this to others.

To address this, the lead researchers utilized the process of memoing regarding developing themes, which allowed for adjustments to any identifiable biases. Following the recommendations of Mauthner and Doucet (2003), the lead researchers set aside designated times during the data collection process to reflect deeply on the data analysis procedures. Consequently, throughout both the collection and analysis phases, the researchers consistently assessed how their personal predispositions might have subtly influenced the interpretation of the data. Further details on the lead researcher’s positionality are provided below.

Most notably, the lead author disclosed to participants their personal history as a caregiver in a private capacity, as well as their professional experience as a certified nursing assistant (CNA). Therefore, the lead researcher was, in some respects, an insider, having experiences that overlapped with those of the participants. This perspective offered distinct advantages, fostering a sense of comfort among participants and encouraging them to openly share their lived experiences with the researcher. Given the often emotionally demanding nature of the interviews, this rapport likely enhanced data quality, even though data was collected via telephone rather than in-person due to the pandemic. However, this insider status carries the risk of potential biases during analysis, as the researcher’s own experiences could unintentionally emphasize or diminish certain emerging themes. While this challenge exists across different research designs, the implementation of inter-rater reliability served as a vital check to mitigate such personal inclinations. Therefore, inter-rater reliability was strategically employed to counteract the possibility of introducing biases.

## Findings

Our analysis of the construction of Aduhelm identified four prominent themes. First, participants addressed ambiguous efficacy and unclear effectiveness of Aduhelm: *Aduhelm’s Ambiguous Efficacy*. Second, participants highlighted ambiguity about Aduhelm’s side effects: *Ambiguity about Aduhelm’s Side Effects*. Third, participants discussed the cost and accessibility of Aduhelm: *Aduhelm, Cost, and Accessibility*. Lastly, the fourth emergent theme underscores the sentiments of mistrust and burnout addressed by participants, as well as additional consequences of the Aduhelm controversy: *Consequences of the Aduhelm Controversy.*

When asked about Aduhelm, most caregivers knew about the drug by name and many more had heard about the controversy. There were 3 caregivers who had never heard of it at all and so those questions were mostly skipped as very little information could be gleaned. Caregivers who knew of Aduhelm relied on narratives presented by various stakeholders, including the pharmaceutical industry, advocacy groups (e.g., the Alzheimer’s Association), the FDA (The Food and Drug Administration), the subreddit r/Alzheimers, and medical professionals to aid in the decision-making process. However, these narratives were often inconsistent or contradictory, with some stakeholders supporting the drug while others expressed ambivalence or concern.

### Aduhelm’s ambiguous efficacy

Despite an informational environment that promoted confusing and contradictory messages, some caregivers in our study took proactive measures by researching medical studies on their own. Hence, it is important to note that these caregivers are not trained in medical research nor sifting through the complicated process of scientific investigation more generally. For instance, while conducting her own investigation online, Ella became keenly aware of the fact that Biogen’s clinical trials were inconclusive, despite the claims from Biogen.

We did some research. The research doesn't show you know, what they (Biogen) wanted to show and so we were just like, forget it. Don't even bother. I don't think we would do anything differently, especially with the controversy that's surrounded it (Aduhelm) and still, you know, causing the FDA headaches. (Ella—Caregiver)

The contentious debate between various stakeholders concerning Aduhelm’s efficacy significantly “muddied the waters” regarding Ella’s decisions about its use. Ella became weary once she believed the findings contradicted what pro-Aduhelm voices proclaimed. Additionally, the controversy itself regarding efficacy reflects the broader implications from influential institutions like the FDA. The perception that Aduhelm posed challenges—"headaches”—for the FDA led many caregivers to question the drug’s promise as well as the authority of the FDA.

Medical professionals mirrored this sentiment and criticized Aduhelm’s efficacy while scrutinizing Aduhelm’s clinical trial data—information that they conveyed to caregivers and patients. Their assessments varied, with some noting the drug’s limited efficacy, while others believed it had promise, yet others suggested Biogen exaggerated the positive results or that findings were statistical anomalies. For example, Clifford, a neurologist, speculated that the results could be a fluke or a statistical artifact rather than a genuine, clinically significant effect.

I’m not super convinced that the effect is even real. It was just in one of the, you know, one of the two trials. I have suspicions that it may be kind of a fluke. There's going to be more trials on other anti-amyloid therapies coming out later this year. So I think that's what we're waiting on to see if there's more evidence that this is an approach that works or not. (Clifford—Neurologist)

Additionally, discussions within the subreddit, r/Alzheimers highlight the controversy surrounding Aduhelm and narratives regarding limited efficacy. For example, one redditor below asks the subreddit via a post about the details of the drug, inquiring about getting access, and expressing uncertainty to the community about the FDA approval process. Another redditor responds and argues the drug is based on a flawed theory about Alzheimer’s disease.

Has anyone had any luck getting access? The rollout since FDA approval has been pretty spotty, and details very murky (Redditor 1—Original Poster).

Do keep in mind that there is so far no significant evidence that Aduhelm actually works, and may well be based off of a completely flawed theory of Alzheimer’s causation: give https://www.sciencehistory.org/distillations/podcast/the-alzheimers-copernicus-problem, a listen. (Redditor 2).

As noted above, the online discussion between the two redditors illustrates the prevailing uncertainty regarding Aduhelm’s efficacy. The exchange also underscores the importance of online communities for understanding medical decisions, as the original poster (OP) expresses apprehension after discussing the drug and hopes to find answers from the subreddit. Additionally, the comment from the second redditor reveals how diverse sources of information, including podcasts in this case, shape caregivers’ perspectives on different treatment options.

### Ambiguity about Aduhelm’s side effects

In addition to doubts about Aduhelm’s efficacy, caregivers expressed concerns about the drug’s side effects, or as many noted, “the cure was worse than the disease.” A crucial aspect of side effects for these caregivers is the desire to ensure a high quality of life for their family members, many of whom were/are near the end of life, or the trade-off between side effects and amelioration of symptoms regarding any medical intervention. While a few caregivers actively sought to have family members begin treatment on Aduhelm, the majority expressed apprehension about side effects considering conflicting narratives about the drug. If they believed that Aduhelm would not substantially enhance their family member’s condition, then the potential side effects were deemed unworthy of consideration. Likewise, many caregivers felt that using the drug might further aggravate the difficulties faced by both the individual with the disease and their caregivers. The statement below from Chet underscores this sentiment:

At this point, I'm trying to make her life as pleasant as possible and whatever little happiness she could have, but, on Aduhelm she wouldn’t really have a better quality of life, in my opinion. (Chet—Caregiver)

This finding supports previous research from Shoemaker and Ramalho de Oliveira (2008), highlighting the concerns about bodily effects of medications and weighing the pros and cons. In line with the data about efficacy, caregivers were informed about side effects through a variety of informational sources, becoming aware of potential side effects and other concerns related to the drug. Many consulted doctors and other medical professionals to discuss whether Aduhelm was appropriate for their family members. The sentiment expressed by a neurologist below illustrates this cautious approach and how it can sway caregivers.

The big thing is brain bleeds. You don't need sort of another insult on top of that. If somebody were to have a hemorrhage, that in and of itself is going to cause dementia. The majority of people that I know that are working with, you know, the general population of Alzheimer's, aren't prescribing it. (Miles—Neurologist)

A redditor posted a similar sentiment regarding Aduhelm and the risk of side effects. They also address concerns about limited efficacy and success.

I think the side effects are of a concern enough that I’m not sure it’s a good idea especially given how little reason there is to think it helps. Patients and families are often drawn to potential treatments and don’t put enough weight on side effects. (Redditor 3)

Both caregivers and medical professionals were aware of the controversy surrounding Aduhelm’s approval. Evaluating the treatment involves balancing efficacy and safety; however, significant amounts of uncertainty regarding Aduhelm’s side effects dramatically shifted the decision-making process. Medical doctors acted as powerful arbiters in the doctor/patient interaction and shared concerns about the drug’s questionable efficacy and potential side effects.

### Aduhelm, cost, and accessibility

Initially, Biogen set Aduhelm’s annual cost at $50,000, intending to have the drug covered by Medicare (Liu, 2021). Policy makers expressed considerable concerns that the drug could potentially strain Medicare financially by adding sizable costs from the thousands of older Americans affected by Alzheimer’s disease. At the beginning of this study, it remained uncertain whether Medicare would cover Aduhelm and was available only to those who could afford to pay out-of-pocket or had private insurance that included coverage, which was rare (Haddad et al., 2022).

Given the high cost, most caregivers found the treatment unaffordable or did not consider it worth the financial strain. Caregivers were mindful of how their social position could limit access to certain healthcare options. However, even when aware of their own access to financial resources, participants concluded that the drug was not for them. For instance, Nina below mentions that Aduhelm was not suitable for them, alluding to their social class position, yet also acknowledged that they have discretionary income.

I’d rather use the 50k for other life experiences. Let's just go on a vacation or something, you know? I mean, I don't know, just especially if it's not really going to do that much and it might cause more problems. Like that's not even financially feasible for what little help it might give you. But yea, that might be for people who have, like, way too much money and are just willing to try anything at this point. (Nina—Caregiver)

The above passage illustrates the caregiver’s preference to allocate funds toward activities they believe would more effectively enhance the quality of life for their family members, rather than rely upon a treatment supported by the medical model. Nina’s statement also highlights a belief shared by many caregivers, that the treatment of Alzheimer’s disease and challenges of caring are best tolerated via lifestyle adjustments to improve quality of life rather than cognitive functioning—in this case taking a vacation.

Medical professionals were similarly cognizant of the drug’s cost and the barriers it created. The medical professional cited below discusses the high expense of the drug and expresses feelings of guilt and apprehension about prescribing it to patients who lack sufficient insurance coverage.

And I’m not sure what to think of it, especially that it's that expensive. So it's a monoclonal antibody. The very expensive kind of drug. I think I would have a lot of reservations in doing so, especially if the patients we are seeing are underinsured or if their insurance doesn't cover this kind of therapy, because it's also like. The outcomes weren't very approved in a very strong way. (Dinah—Medical Resident)

Similar sentiments about the high cost of Aduhelm are echoed on the subreddit, r/Alzheimers. In a discussion about the drug, one redditor comments on its expensive nature and suggests that Biogen could reduce the cost to make it more accessible. Further emphasizing the financial burden, the same redditor mentions their personal struggle to afford the drug even going as far as lowering the temperature in their home during the winter to manage the expense.

I’m having to keep my thermostat at 63 this winter in part due to Biogen and Aduhelm. If there was clear and compelling evidence that it wasn't just expensive snake oil, I could comfort myself that people would live long enough to die of something that kills quicker and more mercifully than Alzheimer’s, but I don't even have that. (Redditor 4)

Facing intense scrutiny over their price tag, Biogen eventually reduced the annual cost to $28,000, an amount still prohibitive for most (Haddad et al., 2022). In April 2022, CMS issued a Coverage with Evidence Development (CED) decision: Medicare would cover Aduhelm only for patients enrolled in approved clinical trials. These trials could be sponsored by Biogen, NIH, or academic partners, but CMS itself was not conducting them. This development introduced a new consideration for both caregivers: participation in the clinical trial meant the medication could potentially be received for free or at reduced cost. However, there was a significant caveat as due to the double-blind nature of clinical trials, participants would have a 50/50 chance of receiving Aduhelm, with no way of knowing whether they were receiving the drug or a placebo. While many caregivers and medical professionals remained ambivalent about Aduhelm, there was one participant which their family member was currently participating in a clinical trial funded. In their case, the cost of the drug was covered to encourage participation in the clinical trial, although it remained uncertain whether the family member was receiving the treatment due to the trial's design. Despite this uncertainty, the caregiver observed and believed that their family member's condition was improving. The thing is, she's been approved and she's getting it and there's no adverse effects. It's possible it's just a placebo. But she says she feels better after she gets it. They're still, you know, participants in a research study, technically. (Sarah—Caregiver)

We find that the fact that at least one caregiver had a positive opinion about Aduhelm further speaks to a degree of ambiguity. As discussed in this section, both caregivers and medical professionals were cognizant of the high cost associated with Aduhelm. The lack of clear justification for this cost and the fact that many medical doctors echoed concerns about the drug’s expense added complexity to the decision-making process. Initially, Aduhelm’s prohibitive price made it inaccessible for most caregivers. Eventually, the drug became available through clinical trials, but this only provided a “chance-based” opportunity to receive the treatment due to the trial’s randomized nature.

### Consequences of the Aduhelm controversy

When participants discussed Aduhelm and the controversy surrounding its approval, participants tended to reflect negative narratives about the U. S. healthcare system in general, fostering a sense of mistrust and disillusionment with the status quo. This section aims to describe these attitudes. Drawing from Williamson and Bigman (2018), medical mistrust is the lack of trust in medical institutions, healthcare providers, treatments, or the healthcare system, often stemming from concerns about the motivations and integrity of institutions. For example, one caregiver expressed a general skepticism toward the pharmaceutical industry, citing concerns over side effects.

You know, we just have a problem with medications like that (referring to Aduhelm). When there's like you see the ones on the commercial and it says, Oh, it's going to help your eczema, but it's going to cause, you know, like flatulence and extreme diarrhea, you know, like kidney stones and ulcers like, come on, really? I'd rather just have the eczema on the cheek. Well, when it causes all these extreme side effects that are like sometimes worse than the original problem, like why? Why are we even doing this? (Nina—Caregiver)

Nina articulates a broad concern about medications, which they specifically associate with Aduhelm. This viewpoint reflects a deeper sense of medical mistrust within the healthcare system, and practices by the pharmaceutical industry influencing attitudes toward Aduhelm more directly and perceived “extreme side effects.” Other caregivers began actively questioning the legitimacy of powerful stakeholders within the health system reflecting a critical stance toward the competence of those who influence decisions about healthcare and treatment options.

And it seems like they’re still, like, fumbling around trying to, you know, get to the root cause. (John—Caregiver)

The term “fumbling around,” used by John above, is in reference to a discussion about researchers and pharmaceutical companies. It vividly conveys a lack of trust in authority figures and casts doubt on their legitimacy. Aduhelm originally gave caregivers hope that there was a path to improve the caregiving experience and their family member’s disease. However, due to the sense of uncertainty regarding the medical establishment, they found themselves lacking clarity and guidance about how to proceed. Unfortunately, this aggravated feelings of hopelessness about engaging with the health system. For instance, one caregiver below expresses a sense of hopelessness, exhaustion, and desperation when asked about Aduhelm.

I honestly have been so overwhelmed. It's really hard to find out what we should do, you know. But yeah, I just…I'm so overwhelmed that a lot of things went by the wayside. And then it's like, well, what’s the point? What's going to come from it? (Aretha—Caregiver)

As noted by numerous caregivers and medical professionals, the controversy surrounding Aduhelm also strained the doctor-patient relationship. Caregivers were given hope by advocacy groups and pharmaceutical companies that Aduhelm could help, but they encountered doctors who, due to concerns about the drug’s safety, had little to offer in terms of treatment. Bessie notes this sentiment in the passage below and shows how the controversy surrounding Aduhelm stresses the doctor/patient interaction.

I got very short with his neurologist, very rude with them toward the end, [they said], ‘why are you so keen to see me? I told you what the problem is, and there's nothing more I can do for you.’ It was very, very frustrating. (Bessie—Caregiver)

The statement from Bessie above underscores the sense of momentary hope stemming from various narratives about Aduhelm in contrast to the frustration with a medical professional who is unable to provide relief. Despite her doctor not being at fault, Bessie directs her frustration toward their doctor. Consequently, there exists a disconnect between differing voices within the health system: the pharmaceutical and advocacy space, which seemed to promote a sense of hope to caregivers, versus the trepidation of medical professionals.

Medical professionals substantiated the taxed doctor-patient interaction as well as frustration with the predominantly for-profit nature of the American health system and the pharmaceutical industry. For example, Dinah, a medical resident, noted that many of their colleagues were concerned that financial motivations primarily drove Biogen’s push for approval.

These are very informal conversations with my coworkers (about Aduhelm), you know. And I do recall the kind of conversations where they emphasize how money-driven healthcare is and how profit driven it is, and how sometimes it comes at the expense of the patient. (Dinah—Medical Resident)

As highlighted above, some medical professionals view the Aduhelm controversy as indicative of the profit-driven motives within the U. S. healthcare system. This puts medical professionals like Dinah in a difficult position. On the one hand, Dinah wanted to support their patients and reduce stress on caregivers. On the other hand, they were cognizant of the issues inherent within the institutions to which they belong, which creates reticence toward prescribing.

Other medical professionals pointed out perceived conflicts of interest within the medical field, raising concerns about how these might have influenced the drug’s approval. These doctors discussed the influence of powerful stakeholders who lobbied government agencies to either approve or expedite the approval process for Aduhelm. The medical professional below alludes to these issues, addressing the role of lobbying in the drug’s regulatory journey.

I can kind of see the side of the Alzheimer's disease patient advocates and the patient groups, they want all possible medications to be made available as soon as possible so that people who are willing to try the medication, have the option to do so. And so I'm thinking that might be part of the reason, that there was the pressure to approve this medication. (Clifford—Neurologist)

Narratives expressing mistrust within the medical system were echoed by redditors on social media. One redditor, for instance, articulates concerns about a conflict of interest involving the Alzheimer’s Association and criticizes the perceived profit motives of Biogen.

This is what evil looks like. Last summer, with pressure from the Alzheimer's Association and against the recommendation of their advisory committee, the FDA approved Biogen's new Alzheimer's drug, Aduhelm. Besides not being effective (the drug has failed to significantly reduce AD symptoms in clinical trials), it has some very unpleasant side effects, and a price tag around $28,000/year. Biogen (who is one of the Alzheimer's Association's biggest donors) stands to lose a LOT of profit on this decision, and the Alzheimer's Association is now pushing back against CMS. I think pushing wildly expensive, ineffective drug on a vulnerable population that is desperate for ANYTHING that can help them is nothing short of predatory. (Redditor 5)

The frustration and alienation expressed by redditor 5 above is palpable in much of the subreddit as other redditors express similar comments. Such remarks underscore the mistrust among some members of the online community toward powerful entities in the healthcare system. Of course, we should note that these statements are anonymous on Reddit, so the redditors face fewer consequences for what they say. However, this can also be explained as a form of “truth serum” considering how they feel about Aduhelm, since they are not affected by social desirability bias and other social pressures and norms related to politeness common in face-to-face interactions (Bergen and Labonté, 2020).

In summation of the findings section, caregivers articulated a pressing need for effective treatments, yet they faced uncertainty often grappling with the trade-offs between efficacy, safety, and cost amidst a deluge of information from various stakeholders, often at odds with each other. As noted in the last section about consequences of the controversy, the sense of ambiguity fueled medical mistrust, burnout, and stressed the doctor/patient interaction as many caregivers were led to believe that Aduhelm had promise as a cure, only to find out that the drug was inadequate. Medical professionals substantiated claims from caregivers, expressing skepticism about the drug’s approval process and its reported benefits, often citing conflicts of interest and a profit-driven healthcare system as significant concerns. This sense of medical mistrust from caregivers and to a lesser extent, medical professionals, was further echoed in online discussions where anonymity allowed for candid discussions about the drug’s efficacy and the ethical implications of its promotion and use. Overall, the controversy surrounding Aduhelm not only influenced individual decision-making but also reflected broader macro-level concerns about medical mistrust as well as the integrity of the US health system. For additional quotes of pertinent themes (see Table 3).

**Table 3:** Themes and additional quotes.

Themes	Additional quotes
*Aduhelm’s Ambiguous Efficacy*	Like what could be a change in the medication approach? I think it’s a lower priority than the like…What is her hourly care situation during the day when people aren’t around? The research does not show you know, what we wanted to show and so we were just like, forget it. Do not even bother. (Ella) From what I’ve read when the decision came out for it to be FDA approved and I think it was even an accelerated approval, it wasn’t even the regular, rigorous process that takes years. A lot of the studies that were considered to be like evidence for its effect were. Poor had poor design. Even analysis was a bit you know, you can always make the data look better depending on what you want your outcomes to be. And in the case of those studies, it was pretty clear that it was embellished a lot. And so I’m not. But at the end of the day, the FDA approved it. (Mary) I think the manufacturers, Biogen and, um. From what I’ve seen, that if it does not really work, it does not do the job that it is supposed to do, which I guess is to remove the proteins in the brain or stops them from developing. They do not know. (Charlie) And the other thing, the whole primary and the reason that we chose not to move forward with it for my brother. And they felt that this drug, it may be too late for any benefits to my brother. (Natalie)
*Ambiguity about Aduhelm’s Side Effects*	I mean, it’s nice to know it’s out there and if it does not make a difference and the doctor recommends it, but there’s also side effects. That’s also good to know, too, because I do not think there’s so much going on there. And it’s it’s really complicated because, you know, side effects can be bad. (Nina) They were causing, you know, bad physical problems. And there just was. And then they, you know…some of the doctors did not react well and you know, it just was not. We just. Yeah, it just really made us leery of getting involved, and we just decided at some point like, let us just, you know, we have got all the medications that are helpful and we really do not want to get, you know, this has been very painful. We do not really want to get very mixed up and you know, and the clinical trials that we can avoid it. (Harvey) But even if you remove the controversy around the post-hoc analyses that Biogen presented us and take them at face value, look at the marginal improvements reported in cognitive function. This drug is not curing AD. Not even close. Is one or two points on a cognitive assessment worth the price tag and the notably severe side effects? Of course not. (Redditor 12)
*Aduhelm, Cost, and Accessibility*	The driving force behind Aduhelm is clearly NOT the well-being of persons with AD - it’s money. Biogen stands to make billions on the drug and the Alz Association will be a beneficiary of these profits. Profits made on the hopes and desperation of our most vulnerable population and their loved ones. I hope that 1 day a drug will come along to dramatically improve or even cure AD. This is not that drug. (Redditor 10) And we all just came to the conclusion that it was. Just not something for that we wanted to kind of put any energy into. We you know, relied on people like Jason Karlawish I think that’s his name. Um, sort of who was like, you know, like, I’m not advising my patients to take it and so, like, yeah, we just we said no to it. We asked the doctors, I think they were like, uh whatever you want, really (Ella).
*Consequences of the Aduhelm Controversy*	I think, um, you know, it was a little unorthodox for the approval to be, to be done it was a little bit accelerated. But, um, yeah, it’s just kind of, it’s just a confusing mess. (Clifford) That’s right, greed is controlling Biogen’s decision making. They might hurt themselves even more by pushing Aduhelm. (Redditor 8) They took her to a neurologist who convinced her that there was nothing wrong. And so for about a year, no treatment was given. And she kept deteriorating. And she was very, you know, becoming really, really upset. And so then my dad had a friend who had a son who who worked in medicine. And my dad went to him and they ended up getting her proper treatment. So I think I think some of the early signs were not because she was so young when she started really science. I think some doctors do not take it seriously. (Sarah)

## Discussion

Building upon the connection between the findings in this study and the extant sociological literature, we formulate a definition for the term, pharmaceutical ambiguity to be used for future study. Pharmaceutical ambiguity is a sense of doubt toward individual pharmaceutical treatments arising from various social forces including incomplete or conflicting scientific evidence, as well as a diverse media landscape in which individuals receive contradicting messages from diffused mediums, advocacy groups, and consumers themselves. Pharmaceutical ambiguity is also subject to the macro-level forces of pharmaceuticalization and the ubiquitous practice of direct-to-consumer pharmaceutical marketing in that pharmaceutical intervention becomes the dominant approach to treatment. While pharmaceutical ambiguity may share features with established sociological concepts like medical uncertainty and medical mistrust, we believe it captures a unique facet of the social construction of a particular drug.

**Figure 2:**
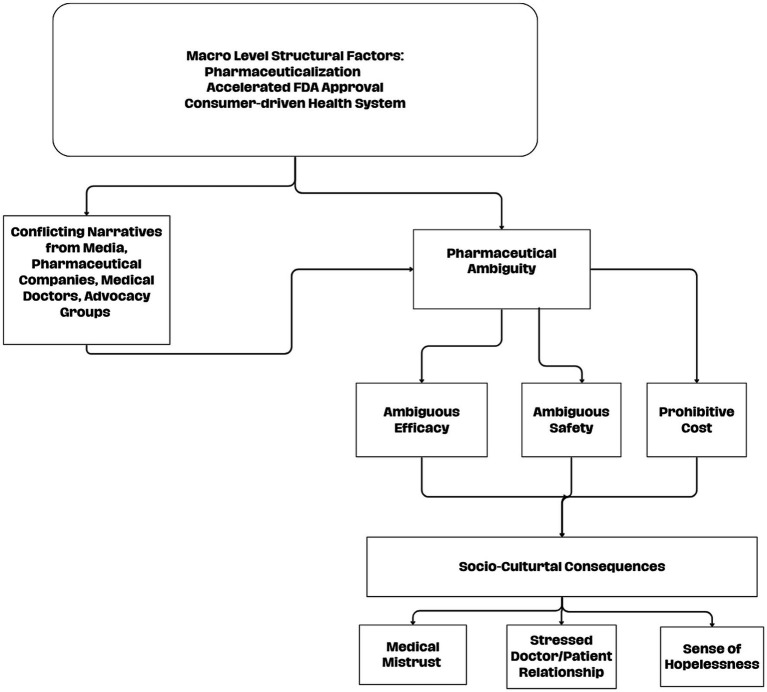
Pharmaceutical ambiguity process.

Pharmaceutical ambiguity occurs when contradictory narratives from legitimate, powerful, and authoritative voices foment confusion, not about medicine in general, but about a specific pharmaceutical. It happens not simply because of lack of knowledge (medical uncertainty) or from a judgment about institutions (medical mistrust), but from the presence of authoritative yet contradictory narratives about a drug’s efficacy, safety, and overall legitimacy. In the era of consumer driven medicine, individuals must decide whether to trust or doubt these authorities. Hence, distinguishing pharmaceutical ambiguity from related constructs shows the ways in which ambiguity may potentially affect adherence as well as the doctor–patient interaction. This makes it a useful theoretical concept for understanding emerging controversies around future novel treatments, where uncertainty is exacerbated by various powerful discourses and the ways in which these social phenomena affect various aspects of the health system. We believe Aduhelm may serve as a case study to identify the social processes affecting pharmaceutical ambiguity as competing narratives about the safety and efficacy of the drug complicated caregiving, creating unease toward pharmaceuticals, and strained the doctor-patient interaction. For a visual representation of the pharmaceutical ambiguity process, see Figure 2.

Regarding the first research question about attitudes toward Aduhelm—pharmaceutical ambiguity led many caregivers to pause or reconsider the decision to use the drug for their family members, highlighting the critical role of clear and trustworthy drug approval processes in treatment decisions. Medical professionals also expressed skepticism, and this influenced the doctor/patient interaction, which was often tense, and plays a large role in adherence to pharmaceuticals (Conrad, 1985). Participants waded through a flood of information from various sources, some within the medical model, others outside of it. Many caregivers expressed a desire to turn away from a medical intervention, instead prioritizing improved quality of life without medicine or pharmaceuticals. Meanwhile, other caregivers asserted that the drug was not developed with people from their social position in mind and felt a sense of apathy. Regarding the second research question, data from this study underscored a broader mistrust of the pharmaceutical industry, regulatory bodies, medical professionals, and advocacy groups, as study participants critically discussed the implications of the drug’s contentious approval.

These novel findings provide theoretical and substantive implications for sociological research. First, we find that pharmaceutical ambiguity may extend to a general sense of mistrust among caregivers. Caregiving is burdensome, and many caregivers are desperate for any respite. Amid the promises of pharmaceuticalization and the increasing reliance upon pharmaceutical intervention in everyday life, caregivers develop a sense of hope from the research regarding Aduhelm, only to have their hopes dashed, creating further disillusionment.

Second, medical professionals are on the front lines of challenges stemming from pharmaceutical ambiguity and may struggle to adequately address patients’ needs and concerns. Currently, there are high hopes within the medical community of a breakthrough in terms of similar pharmaceuticals—see Lecanumab (Woloshin and Kesselheim, 2022). However, the degree of pharmaceutical ambiguity created by the Aduhelm controversy may prove long-lasting and reduce adherence for new drugs.

Third, the rise of pharmaceutical treatments for Alzheimer’s disease and the resulting ambiguity may widen lingering health disparities. Upper-class caregivers, utilizing greater cultural health capital and financial resources (Shim, 2010), are more likely to positively engage with doctors, use medical information proactively, and potentially, navigate pharmaceutical ambiguity with more confidence. In contrast, lower-class caregivers may struggle not only to afford treatments but also with utilizing relevant biomedical knowledge and navigating the health system, making it harder for them to cope with pharmaceutical ambiguity (Simoni and Hilfiker, 2024). Thus, judging from previous literature, we suggest other researchers could further explore this question by looking at the role of social class or other social statuses on the impact of pharmaceutical ambiguity.

Fourth, the approval of Robert F. Kennedy (RFK) as the Secretary of Health and Human Services in 2025 may exacerbate pharmaceutical ambiguity in the coming years and decades. The fact that a person with a well-documented history of anti-vaccine (Yang, 2025) and anti-pharmaceutical activism (Wise, 2025) s leading one of the most powerful public health agencies in the United States government implies that individuals, including caregivers, may doubt the information their government is giving them. Furthermore, budget cuts to public health agencies inflicted by the Trump administration may hamper the ability of the federal government to reliably test, research, and communicate information about pharmaceutical and medical technology.

All academic studies face limitations, and this one is no different. First, the pandemic had a powerful impact on the ability to collect data. Unfortunately, using face to face interviews was nearly impossible considering the virulence of the Covid-19 virus. Although this limitation was a barrier to the collection of data, the use of Reddit as a recruitment tool worked well to collect rich data for this project. Second, results do not speak to generalizability, nor is that the intention. When it comes to Reddit, it is known for having a slight bias toward middle-class, white, young, and male users. This idea comes from the fact that several popular subreddits tend to receive comments from these groups to a somewhat greater extent. However, our sample does not follow the general pattern for Reddit users as caregiving related concerns tend to affect older people, and thus, our sample includes more older individuals, indicative of the population we are interested in studying (see Tables 1, 2).

Finally, this study highlights the impact of pharmaceutical ambiguity surrounding Aduhelm on caregivers, emphasizing the challenges posed by conflicting narratives about the drug’s efficacy, safety, and cost. Our findings reveal that these controversies contribute to heightened pharmaceutical ambiguity, skepticism, and medical mistrust among our participants. Addressing these concerns is crucial to improve adherence, enhance patient-caregiver interactions, and maintain positive attitudes about the healthcare system amidst a heavily consumer-driven and pharmaceuticalized health system.

## Data Availability

The raw data supporting the conclusions of this article will be made available by the authors, without undue reservation.
