# Supplementary material for: The social construction of Aduhelm in the context of pharmaceutical ambiguity: exploring narratives from informal caregivers, medical professionals, and redditors on r/Alzheimers
Source: Front Sociol. 2026 Jan 5;10:1636160. doi: 10.3389/fsoc.2025.1636160 (PMC12812559; doi:10.3389/fsoc.2025.1636160)
Supplement: Supplementary file 1 [file Data_Sheet_1.docx]

Appendix: Semi-structured interview guides

Semi-Structured Interview Guide for Interviews: Caregivers

- Building Rapport and Introduction (2 mins)
  - Interested in Alzheimer’s caregiving because it is understudied sociologically and hope to find ways to help caregivers. Understand different ways to frame caregiving and treatment. Hopefully identify additional forms of support for caregivers, etc.
- Background
  - Could you provide a bit of background about your caregiving experience? Who do you care for, how long?
- Noticing Symptoms and other’s responses (5 mins)
  - When did you start noticing symptoms?
    - How did others respond?
    - How did you respond?
  - How did things change for you, the person, and other caregivers post diagnosis?
    - Did they and you continue to remain socially active?
- Challenges and Informal Caregiving Experience (5 mins)
  - What are some of the biggest challenges and barriers to caring for this person?
  - Who does most of the caregiving for the person in your family?
  - Do others help with the caregiving process? If so, how?
  - How does caring for them affect your life?
- Health System, Pharmaceuticalization, and Attitudes about Aduhelm (15 mins)
  - At what point did the person see a doctor or other health professional?
  - How effective was the care they received?
  - How you feel like you get along with the doctors or medical professionals?
    - Did you and they feel listened to by medical professionals?
    - What is your general opinion of the medical care you received?
  - What kind of medical care have they received since? In home care; assisted living; etc.
  - What forms of treatment does the person currently take?
    - Do you feel they are working?
  - What forms of treatment do you know about?
  - Are you familiar with Aduhelm/Aducanumab?
    - Do you have any concerns or reservations about it?
    - How does the approval process by the FDA influence your opinion?
    - How do you feel about side effects and medications?
    - Have you spoken with a medical profession about Aduhelm?
    - Would you feel happy if your loved one received this treatment?
- How did the pandemic affect the caregiving experience? (5 mins)
- Resources for support (5 min)
  - Where do you get information about Alzheimer’s or dementia?
  - How often do you any resources for support?
- How have you used the subreddit r/AlzheimersClosing
  - Don’t forget about mailing address
  - Any other questions for me

Semi-Structured Interview Guide: Medical Doctors

- Rapport Building Questions and Background
  - Let me begin by telling you a little bit about myself…
  - Interested in factors that affect building trust, compliance, and burnout when diagnosing and treating those with AZ.
    - It is understudied sociologically, and I think it can help with policy going forward and inform best practices.
    - How does the approval of Aduhelm affect this process?
- First, could you provide a little bit of background about your practice and general experiences with dementia or AZ patients?
- How do caregivers generally interact with you?
  - Are you hesitant to give a diagnosis considering potential stigma?
  - Is this interaction more challenging than others?
  - Can you recall the most difficult patient/caregiver you have encountered?
- Do you need to prepare yourself mentally and emotionally for these interactions?
  - How does the interaction make you feel?
- Have you received any pushback from patients?
- What are the factors that lead to burnout amongst medical doctors dealing with AZ patients?
  - What techniques do you use?
  - What resources do you use?
  - What resources do you need?
- Do you recall if you were trained to deal with dementia or AZ?
- Are you familiar with Aduhelm/Aducanamab?
  - Do you have any concerns or reservations about it?
  - How does the approval process by the FDA influence your opinion?
  - How do you feel about side effects and medications?
  - Describe the biggest challenges in managing patients’ Alzheimer’s disease
- Closing
  - Don’t forget about mailing address
  - Any other questions for me
